# Status of Fishery Discards and By-Products in Greece and Potential Valorization Scenarios towards a National Exploitation Master Plan

**DOI:** 10.3390/md22060264

**Published:** 2024-06-07

**Authors:** Efstratios Roussos, George Triantaphyllidis, Vassiliki Ilia, Konstantinos Tsagarakis, Athanasios Machias, Leto-Aikaterini Tziveleka, Vassilios Roussis, Efstathia Ioannou, Yannis Kotzamanis

**Affiliations:** 1 Hellenic Centre for Marine Research, Institute of Marine Biology, Biotechnology and Aquaculture 46.7 Km Athens-Sounio, 19013 Anavyssos Attica, Greece; e.roussos@hcmr.gr (E.R.); gvtrianta@hcmr.gr (G.T.); vilia@hcmr.gr (V.I.); 2 Hellenic Centre for Marine Research, Institute of Marine Biological Resources and Inland Waters, 46.7 Km Athens-Sounio, 19013 Anavyssos Attica, Greece; kontsag@hcmr.gr (K.T.); amachias@hcmr.gr (A.M.); 3 Department of Pharmacognosy and Chemistry of Natural Products, Faculty of Pharmacy, School of Health Sciences, National and Kapodistrian University of Athens, Panepistimiopolis Zografou, 15771 Athens, Greece; ltziveleka@pharm.uoa.gr (L.-A.T.); roussis@pharm.uoa.gr (V.R.); eioannou@pharm.uoa.gr (E.I.)

**Keywords:** valorization, discards, fishery by-products, high-added-value biomolecules, hydrolyzed peptides, marine collagen, minerals, fish meal, fish oil, Greece

## Abstract

The valorization of aquaculture/fishery processing by-products, as well as unavoidable/unwanted catches and discards in Greece, is currently an underutilized activity despite the fact that there are several best practices in Northern Europe and overseas. One of the main challenges is to determine whether the available quantities for processing are sufficient to warrant the valorization of discards and fish side streams. This is the first attempt to systematically record and analyze the available quantities of fish by-products and discards in Greece spatially and temporally in an effort to create a national exploitation Master Plan for the valorization of this unavoidable and unwanted biomass. A thorough survey conducted within the VIOAXIOPIO project unveiled a substantial biomass of around 19,000 tonnes annually that could be harnessed for valorization. Furthermore, the production of various High-Added-Value Biomolecules (HAVBs) was investigated and experimental trials were conducted to assess the potential yields, with the collected data used to formulate four valorization scenarios.

## 1. Introduction

The world’s seas and oceans have long been a source of sustenance, providing a vital lifeline for communities that rely on fisheries for their economic livelihoods and nutritional needs. However, the sustainable management of marine resources has become an increasingly urgent global concern, with fisheries’ discards emerging as a prominent issue at the forefront of discussions. The introduction of the landing obligation (LO) in the European Union, a pivotal policy shift in the EU’s management of fisheries [1], has brought renewed attention to the complex challenges surrounding discards and has spurred a global conversation on their avoidance and potential valorization [2,3,4]. The LO, also known as the discard ban, was introduced as part of the European Union’s Common Fisheries Policy (CFP) reforms to address the issue of unwanted catches being discarded at sea. The primary objective of the LO is to promote the sustainable management of fisheries by requiring fishermen to land and account for all catches, including previously discarded species.

Historically, discards, or the practice of returning unwanted or non-targeted catch back to the sea [5], have posed a threat to marine ecosystems and fish stocks. This wasteful practice not only undermines the principles of sustainable fisheries but also hampers efforts to achieve long-term environmental balance. The LO, implemented in various regions worldwide, mandates that all catches, including discards, must be landed and accounted for. This policy shift aims to promote responsible fishing practices, reduce overfishing, and encourage the industry to adopt more selective and environmentally friendly methods [6].

As the fishing industry grapples with the implications of the LO, the need to explore effective strategies for discard avoidance has become paramount [7]. Fishers, policymakers, and scientists are collaborating to develop innovative technologies, gear modifications, and management approaches that minimize bycatch and discards without compromising the economic viability of fishing operations [8]. Moreover, the valorization of discards—transforming them into valuable products such as fish meal, nutraceuticals, or other bio-based materials—presents an opportunity to turn a historically wasteful practice into a sustainable and economically viable solution [9,10]. In this context, a discussion delves into the multifaceted aspects of fisheries’ discards, exploring the challenges and opportunities associated with their avoidance and valorization [6]. Taking into account the latest research, policy developments, and industry initiatives, scientists aim to shed light on the dynamic landscape of fishery management in the era of the LO [11]. The journey towards more sustainable and responsible fishing practices requires a collaborative effort, engaging stakeholders from all sectors to navigate the seas of change successfully [12].

The impact of the LO on discards in European fisheries has been a subject of ongoing research and assessment [13,14]. Several studies and reports have assessed the effectiveness of the LO in reducing discards, and the results have varied across different fisheries and regions. The implementation of the LO has led to changes in fishing practices, gear modifications, and increased selectivity to minimize unwanted catches [15]. However, the extent to which discards have been reduced depends on various factors, including the specific fisheries, the type of gear used, and the adaptability of the fishing industry [14]. The effectiveness of the LO may vary depending on the species and the complexity of the fisheries. There have been instances where compliance with the LO has been difficult, especially in mixed fisheries where several species are caught simultaneously. In some cases, fishermen have expressed concerns about the economic viability of their operations and the potential for increased unwanted catches. Overall, the impact of the LO on discards in European fisheries is a nuanced and evolving issue. While positive strides have been made in some fisheries, challenges remain, and ongoing efforts are needed to refine and adapt fishery management strategies to achieve the overarching goals of sustainability and reduced discards. Continuous research, stakeholder engagement, and adaptive management approaches are crucial to addressing the complexities of discards in the context of the LO.

The EU Scientific Technical and Economic Committee for Fisheries (STECF) reports that even though the LO has been in force for nearly ten years, and the STECF has been assessing Joint Recommendations submitted since 2014, it is apparent that there is little obvious change in fishing practices to avoid unwanted catches [14]. Exemptions are used principally to maintain the fisheries’ status quo rather than as a last resort to cover small, residual unwanted catches. The majority of exemptions are still justified as being to avoid choke situations, yet there is little evidence of such situations occurring. The STECF concludes that despite several research projects (e.g., DISCARDLESS, MINOUW, MedBLand, etc.) showing otherwise, there is no expectation from the sector that improved practices and an increase in selectivity will lead to positive economic returns. Improving size and species selectivity would require considerably larger meshes, which may significantly reduce profitability [11,14]. The urgent need to reduce the biological impacts of bottom trawling in the Mediterranean should be addressed by promoting the adoption of more ecologically sustainable fishing gears through the introduction of more selective meshes or gear modifications [16].

Moreover, waste from the fishery processing industry and retail markets is produced in huge quantities and its valorization can be an emerging biotechnology field, even in an oligotrophic sea such as the Mediterranean, where its biological productivity decreases from north to south and west to east [17,18]. Waste management is a socially relevant issue that has an impact on society and as seafood waste accounts for nearly two-thirds of the entire amount caught, raising serious environmental and economic issues, it is now imperative to address the question of how to dispose of and recycle these pollutants [19].

To solve environmental issues and fully utilize fish side streams, which have significant commercial value, better fish waste management is therefore imperative. In recent years, there has been a rise in interest in finding alternate applications for fish by-products, which has aided in sustainable development and economic expansion. The scope of this paper is to estimate, in a systematic way, the volume of fish by-products and fishery discards (FBPD) in Greece, as well as their potential for the production of high-added-value biomolecules (HAVBs), such as fish meal, fish oil, various forms of collagen, fatty acids, and mineral trace elements, in order to design/initiate a national Master Plan for their exploitation.

## 2. Results

### 2.1. The HAVBs That Can Potentially Be Produced from FBPD

A series of potential products that can be obtained from FBPD have been investigated, aiming to add value to every kilogram of unavoidably caught and discarded biomass while also taking into account category 3 fish by-products (FBP-3, i.e., carcasses and the parts of slaughtered animals that are of low risk and are not intended for human consumption for commercial reasons; for a definition, see [20]) from the processing of fish. In addition, the aim was to develop and assess practices that have been applied in several European countries, after adaptation to Greek conditions. These products can be directed either to human use and consumption, therefore possibly resulting in a higher price, or be used as feed ingredients, depending on the preservation methodology and separation procedures.

Of course, there are many more products (HAVBs) that can be produced, but this study was focused on the products presented in Table 1, depending on the source of fish to be exploited and the way in which it is preserved.

### 2.2. Estimation of Potential Sources of Raw Material for the Production of HAVBs from FBPD

Three potential sources of raw material for the production of HAVBs using FBPD have been identified.

#### 2.2.1. Catches That Are Not Ultimately Sold at the 11 Official Greek Fish Auctions (Hereafter Referred to as Landing Sites)

The estimate is that, on an annual basis, they amount to approximately 722 tonnes, of which 87% is concentrated in three fish landing sites of the Central Markets and Fishery Organization (CMFO): 273 tonnes in Thessaloniki, 114 tonnes in Kavala, and 245 tonnes in Piraeus (Table 2).

#### 2.2.2. Discards

The term discards refers to the percentage of unwanted catches that, after being brought on board, are then discarded at sea due to their unmarketable value. Significant quantities of discards are produced by the artisanal fleet (about 14,500 vessels). However, the fleet operates throughout the Greek territories and it is difficult to integrate the vessels into central planning since the landing points are scattered throughout the Greek territories. The Greek designated ports for the landing of Reg. (EC) No. 1967/2006 and swordfish (SWO) and bluefin tuna (BFT) landing/transshipment number 252. Thus, emphasis should be placed mainly on trawlers (236 vessels in 2022 and 5850 tonnes of annual average discards) and secondarily on purse seiner vessels (224 vessels in 2022 and 1803.3 annual average tonnes of discards). The data show that the mean annual discard estimate summed across all areas and combined for trawlers and purse seiners is 7653.3 tonnes (Table 3). The discarded catch from artisanal fisheries is estimated at 3417 tonnes for the whole country of Greece (see Supplementary Materials); however, although this figure is not negligible as it is scattered throughout the Greek territories, these quantities cannot currently be included in the central planning of their exploitation as the logistics for their collection and concentration into a processing unit would be quite expensive. These quantities could be considered at a later stage.

#### 2.2.3. Category 3 Fish By-Products (FBP-3) from the Processing of Fish in the Commercial and Processing Chain

The analysis showed that there is a potential biomass in the order of 10,504 tonnes (average value for the years 2017–2021) for the production of HAVBs from FBP-3 and the processing of fishery and aquaculture products in the commercial and processing chain. Supermarkets already apply a system of collection and removal of FBP in contrast to fishmongers and street markets; however, they are directed towards compost and biogas production and not towards the production of HAVBs. From the estimated quantities of FBP, there is an amount of 3821 tonnes in the Attica region and 2056 tonnes in the Thessaloniki region (Table 4).

#### 2.2.4. Total Biomass Available for Valorization

If the above sources are classified by geographical area, it follows that the total potential biomass of raw material for the production of HAVBs from FBPD is of the order of 18,879 tonnes on an annual basis. The largest amount is recorded in the region of Attica, where 4524 tonnes per year could be collected and utilized (23.96% of the total), followed by the region of Eastern Macedonia—Thrace (Kavala and Alexandroupolis), where 3747 tonnes can be collected annually (19.85% of the total) and the region of Central Macedonia with 3320 tonnes per year (17.59% of the total), although 255 tonnes of FBP produced in the retail outlets of the Western Macedonia region have also been taken into account. These three areas gather 61% of the potential raw materials for the production of HAVBs from FBPD (Table 5). The highest quantities are available during October (2066.71 tonnes), November (1897.30 tonnes), and March (1717.70 tonnes), and the lowest in July (1075.65 tonnes) and August (1178.33 tonnes).

### 2.3. Assumptions

Taking into account the total potential fishery biomass sources in Greece (Table 5), it is clear that for the creation of the biomolecules of high added value (Table 6), there are many combinations in terms of the available raw material of fishery by-products and discarded fish to be exploited. Some assumptions must therefore be made in order for the solution(s) to be given and become more structured.

First Assumption: In principle, the quantities listed in Table 3 were taken into account.

Second Assumption: All quantities of discarded catches from trawlers and purse seiners will be channeled into the category of potential products of the category “1. HAVBs from fresh or frozen FBPD” from Table 1. All quantities of the catch that are not finally sold at the fish landing sites, as well as those of FBP-3 from the processing of fish in the commercial and processing chain (including supermarkets), will be channeled to the category “2. HAVBs from ensilaged FBPD” from Table 1.

### 2.4. Yields per Production Line and Master Plan Scenarios

The monthly quantities of FBPD in Greece are not negligible and can support the development of a dynamic blue bio-refinery that valorizes the quantities of discarded catches from the three main sources identified, i.e., (i) fish catch that is not sold at the Greek fish landing sites (722.13 tonnes per year), (ii) discarded catches from trawlers and purse seines (7653.3 tonnes per year), and (iii) fish by-products from fish processing in the commercial and retail processing chain (7749.3 tonnes per year).

Therefore, the following four indicative scenarios have been analyzed:

**Scenario A:** Valorize the quantities that will be landed at the fish landing sites of Kavala, Alexandroupoli, Thessaloniki (Nea Michaniona), Piraeus, Chios, and Patras. This is equal to 5656.6 tonnes on an annual basis, which is equivalent to 73.91% of the annual quantity of 7653.3 tonnes.

**Scenario B:** Valorize the quantities that will be landed at the fish landing sites of Scenario A, plus the fish landing sites of Chalkida and Volos. This is equal to 6270.6 tonnes on an annual basis, which is equivalent to 81.93% of the annual quantity of 7653.3 tonnes.

**Scenario C:** Valorize the quantities that will be landed at the fish landing sites of Scenario A, plus the fish landing sites of Scenario B and Cyclades. This is equal to 7016.4 tonnes on an annual basis, which is equivalent to 91.68% of the annual quantity of 7653.3 tonnes.

**Scenario D:** Valorize the quantities collected from unsold catches in the fish landing sites of Piraeus, Thessaloniki, Kavala, Alexandroupolis, Patras, Chalkida, and Volos, as well as those from FBP-3 from the processing of fish in the commercial and processing chain (including supermarkets), which is equivalent to 79.71% of the annual capacity which amounts to 8947.9 tonnes.

As mentioned in the assumptions, the quantities of the catch that are not finally sold at the fish landing sites, as well as those of FBP-3 from the processing of fish in the commercial and processing chain (except those that are traded in the supermarkets that are obliged to follow Reg (EC) 1069/2009 and Reg (EC) 142/2009), will instead be channeled to the category “2. HAVB from ensilaged FBPD” from Table 1.

**Figure 1:**
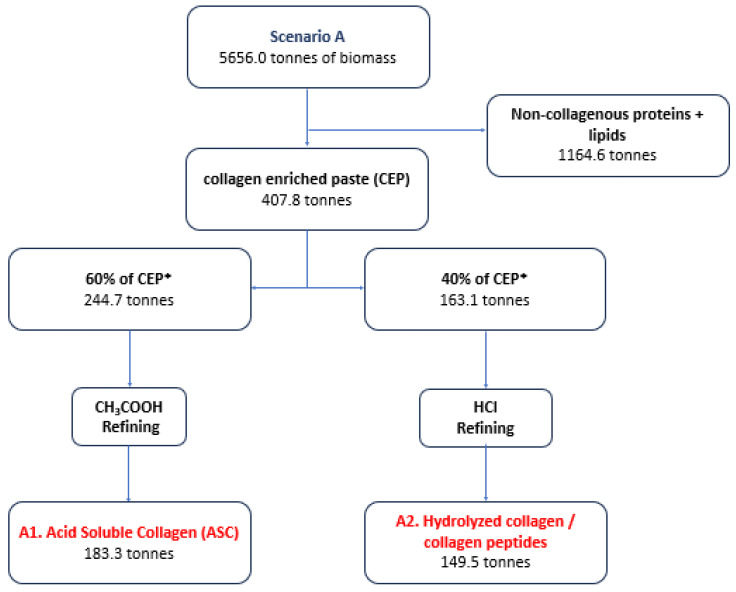
Scenario A for the production of HAVBs based on the utilization of fresh or frozen discards. Quantities in tonnes. * The percentages used are indicative depending on market fluctuation and prices.

**Figure 2:**
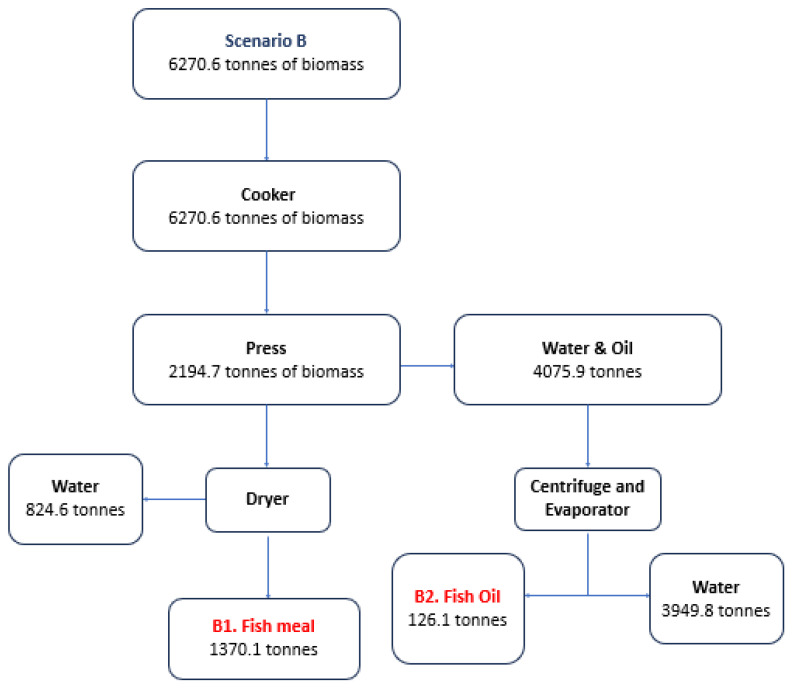
Scenario B for the production of HAVBs based on the utilization of fresh or frozen discards. Quantities in tonnes.

**Figure 3:**
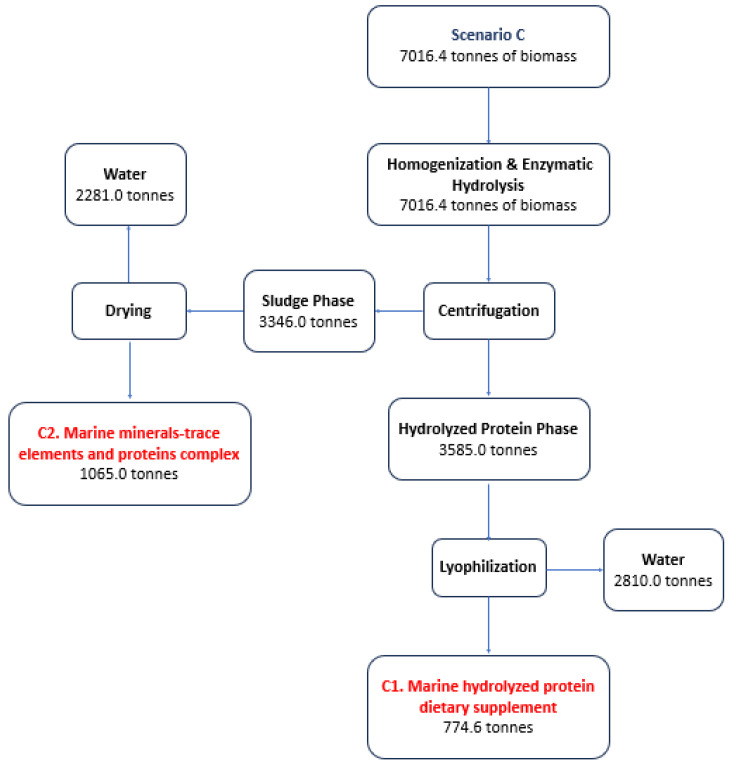
Scenario C for the production of HAVBs based on the utilization of fresh or frozen discards. Quantities in tonnes.

Figure 1, Figure 2 and Figure 3 analyze indicative scenarios from the utilization of fresh or frozen discarded catches for the production of HAVBs. In total, six different products can be produced from four production lines.

In Scenario A (73.91% of the annual capacity), the average monthly biomass is 471.39 tonnes, while the maximum amount is 683.2 tonnes (October) and the smallest amounts are observed in the months of July (239.1 tonnes) and September (243.3 tonnes). In Scenario A, the utilization of the available biomass will be mainly directed to the production of high-value bioactive compounds. Initially, all the available fish biomass will be used to produce collagen-enriched paste (CEP) as it is the first step of the refining process for both production lines. The biomass will be dried, resulting in 4084 tonnes of moisture, and the refining will produce approximately 407.8 tonnes of CEP. Afterwards, 60% (244.7 tonnes) will be directed to produce 183.3 tonnes of A1. Acid-Soluble Collagen (ASC) and 40% (163.1 tonnes) will be used to produce 149.5 tonnes of A2. Hydrolyzed collagen/collagen peptides. The total utilization of dry biomass is calculated to reach about 21.2% (Figure 1).

In Scenario B (81.93% of annual capacity), the average monthly biomass is 522.56 tonnes while the maximum amount is 765.1 tonnes (October), and the smallest amounts are observed in the months of July (256.3 tonnes) and September (256.0 tonnes). Scenario B will prioritize the production of animal feedstuff to harness all of the unutilized fish biomass (6270.6 tonnes), resulting in 1370 tonnes of fish meal and 126 tonnes of fish oil. The valorization of total dry biomass in this scenario rises to 80%.

In Scenario C (91.68% of the annual capacity), the average monthly biomass is 584.71 tonnes, while the maximum amount is 891.5 tonnes (October) and the smallest amounts are observed in the months of July (265.5 tonnes) and September (274.0 tonnes). In Scenario C, fish will predominantly be valorized using enzymatic hydrolysis to produce 774.6 tonnes of C1. Marine hydrolyzed protein dietary supplements and 1065 tonnes of C2. Marine mineral trace elements and protein complexes using a single production line for a total dry matter (DM) utilization percentage of 87.4% (Figure 3).

**Figure 4:**
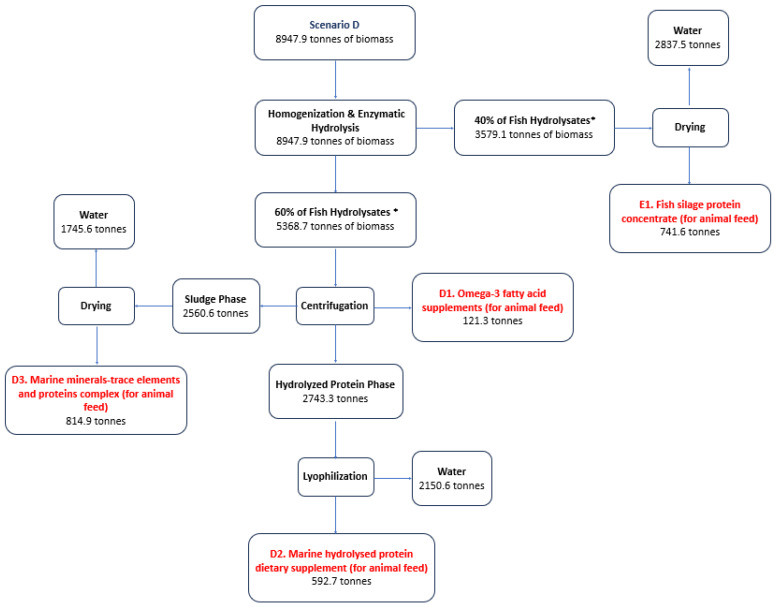
Scenario D for the production of HAVBs based on the utilization of ensilaged biomass from fish catch that is not sold at the Greek fish landing sites (Table 2) and from fish processing in the commercial and retail processing chain (Table 4). Quantities in tonnes. * The percentages used are indicative and depend on market fluctuation and prices.

Figure 4 presents an additional indicative scenario for the utilization of unsold catches, as well as those from FBP-3 from the processing of fish in the commercial and processing chain (including supermarkets), per fish farm area as a potential source of raw material for the production of HAVBs. Three different products result from the separation of the silage fractions and one more from drying the silage without further processing.

Specifically, in **Scenario D**, the quantities collected from the fish landing sites of Piraeus, Thessaloniki, Kavala, Alexandroupolis, Patras, Chalkida, and Volos are used, which amount to 8947.9 tonnes on an annual basis, equivalent to 79.71% of the annual capacity. The average monthly biomass is 566.12 tonnes, while the maximum amount is 638.5 tonnes (April) and the smallest amounts are observed in the months of August (417 tonnes) and February (469 tonnes).

In Scenario D, 60% of the annual quantity (equivalent to 5368 tonnes) is directed to production line D, which concerns the separation of the three phases of silage, resulting in 121.3 tonnes of Ω-3 fatty acid supplements, 592.7 tonnes of Marine hydrolyzed proteins, and 815 tonnes of complex marine metal trace elements and proteins. The remaining 40% of the annual quantity (equivalent to 3579 tonnes) is directed to the silage production line. After drying, 741.6 tonnes of protein concentrate are produced for a DM biomass utilization of 84.6% (Figure 4).

## 3. Discussion

The analysis of the data obtained highlights the significant valorization potential in Greece for the production of HAVBs derived from FBPD. The data categorize the raw material by geographical areas, shedding light on the distribution of available resources and emphasizing the possibility of creating a national exploitation Master Plan. Currently, the available fish discards and by-product biomass is practically unexploited in Greece, in the sense that no HAVBs are currently produced.

The data reveal that the geographical distribution and concentration of the FBPD biomass is primarily located in the regions of Attica, Eastern Macedonia—Thrace, and Central Macedonia, collectively constituting 61% of the total biomass. This concentration suggests that these regions could play a pivotal role in any strategic planning for HAVB production. The abundance of raw materials in certain regions, particularly in Central Macedonia and Eastern Macedonia—Thrace, presents a valuable opportunity for sustainable resource utilization and economic development.

The incorporation of fishery by-products from retail outlets in the Western Macedonia region underscores the importance of considering the entire supply chain in the analysis. This integration brings forth a more comprehensive perspective on the potential biomass available for HAVB production. Evaluating the dynamics of by-product generation in retail outlets can offer insights into consumer behavior, waste management practices, and opportunities for collaboration between different sectors.

The temporal dynamics of biomass availability throughout the year are crucial for planning and operational considerations. The higher quantities in October and November are related to specific fishing seasons, migration patterns, or other ecological factors. Understanding these patterns can aid in the development of adaptive strategies to maximize resource utilization during peak periods.

This study emphasizes the role of utilizing fishery discards and by-products in the context of environmental sustainability and the circular economy. The production of HAVBs from these resources aligns with the principles of minimizing waste and creating value from otherwise discarded materials. Strategies for sustainable harvesting, processing, and utilization must be explored to ensure the long-term viability of such initiatives.

In addition to being safer and more socially acceptable than the collagen isolated from terrestrial sources, several reports indicate that the presence of marine collagen in biomaterials could promote cell adhesion, differentiation, and growth, as well as wound healing [21]. Thus, marine collagen and its denatured and hydrolyzed forms could find applications not only in the food sector but also in health-related sectors, namely in cosmetics, the pharmaceutical industry, and in medical care [18].

The targeted value chains present significant economic interest as the marine collagen market is expected to exceed USD 980 million by 2025, due to the constantly increasing usage of collagen in the cosmetic, food, and beverage industries [22], with fish waste presenting a vast and unutilized source of collagen for these industries [23]. Moreover, the average market value for hydrolyzed collagen could reach a minimum of EUR 16 per kilogram, as stated by Araujo et al. (2021) [24], depending on the production process and product quality.

As for the fish hydrolysates, the market size was estimated at USD 420 million globally in 2019 and it is expected to increase by 4.5% CAGR (compound annual growth rate) in the time between 2020 and 2026 [25]. Marine organisms are an excellent source of bioactive peptides [26], and the isolation of hydrolysates and purified peptides from various fish species by means of enzymatic hydrolysis, acid–alkaline hydrolysis, and fermentation has been excessively studied [27,28].

Regarding food and pharmaceutical applications, enzymatic hydrolysis presents the most suitable method as it produces high-quality peptides without any residual organic solvents and chemicals [29]. Hydrolysates and purified peptides have been reported to prevent various human pathologies, with antimicrobial, antihypertensive, antioxidant and neuroprotective activities being commonly referred to as health-related functions [30].

Considering the experimental yields and the available literature, protein hydrolysates could potentially be a profitable valorization method since the average market value could reach EUR 22 per kilogram for human consumption, as reported by Araujo et al. (2021) [24]. For animal feed and pet food purposes, the global fish protein hydrolysate industry in 2018 produced 39127 tonnes of fish protein hydrolysate in powder form and 5411 tonnes in liquid form for approximately USD 167.94 and 12.35 million, respectively. The main processing method for the industrial production of fish protein hydrolysate is exogenous enzymatic hydrolysis, which claimed 83% of the global market in 2018, followed by acidic hydrolysis at 12% and autolytic hydrolysis at 5% [31].

Along with the annual increase in global aquaculture production, there is a simultaneous need for sustainable aquafeeds that do not rely largely on fish meal and fish oil [32] as the main protein and lipid sources, respectively, particularly for carnivorous fish species. The rising gap between supply and demand has prompted the search for sustainable alternatives, such as discarded fish species and fish processing by-products that have been reported to have high nutritional value by Saleh et al. (2022) [33] and Khiari et al. (2022) [34]. From a nutritional standpoint, fish meal produced from discards and processing by-products have been evaluated for the diets of Atlantic salmon (*Salmo salar*) [35], rainbow trout (*Oncorhynchus mykiss*) [36,37] red drum (*Sciaenops ocellatus*) [38,39], olive flounder (*Paralichthys olivaceus*) [40], Nile tilapia (*Oreochromis niloticus*) [41], kuruma shrimp (*Penaeus japonicus*) [42], juvenile turbot (*Scophthalmus maximus*) [43], red seabream (*Pagrus major*) [44,45], juvenile longfin yellowtail (*Seriola rivoliana*) [46], river catfish (*Hemibagrus nemurus*) [47], and European seabass (*Dicentrarchus labrax*) [48], with promising results for partial and total industrial fish meal substitution. Data from the proximate composition analysis of Mediterranean fish species with relatively low economic value suggest that the nutritional profile is comparable to industrially produced fish meal; therefore, the concept of a fish meal processing chain could present a viable solution for the valorization of discards and fish by-products.

As for fish oil, the global market size is projected to reach USD 2800 million by 2027 [49], with Europe producing approximately 120,000 tonnes of fish oil each year, driven by the increasing demand of the aquaculture industry, which is utilizing 90% of the global fish oil supplies [50]. To satisfy the demand, the global production of oil from fish by-products accounted for 26% of total fish oil production in 2016, and it is expected to increase in volume [51]. For both fishing and aquaculture industries, oils are a significant fraction of processing waste that depends on the fish parts, age, sex, nutritional status, and time of the year [52]. Fish biomass typically contains 2–30% oils and about 50% of the body ends up as by-products from processing operations [53]. In particular, fish oil could be generated from visceral mass, flesh, heads, and tails in varying quantities [54] to satisfy the need for long-chain polyunsaturated fatty acids (PUFAs), including eicosapentaenoic acid (EPA, C20:5, n-3) and docosahexaenoic acid (DHA, C22:6, n-3), that are essential for human health. Since they cannot be biosynthesized, they need to be supplemented through diet [55]. Long-chain fatty acids from industrial fish processing operations can be valorized in multiple markets such as the food/feed and pharmaceutical industries [56]. Taking into account the current average market value of fish oil, which ranges from USD 5.2 per kilogram for animal feed usage up to USD 12 per kilogram for fish oil with high levels of EPA and DHA (28%) [57], and the projected increase in the future, such a processing line could produce a valuable raw material which promotes the circular economy and mitigates environmental issues.

To successfully implement a national exploitation Master Plan, collaboration among various stakeholders, including government bodies, fishing industries, environmental agencies, and research institutions, is essential. Policymakers need to consider the economic, social, and environmental implications and engage with local communities to ensure a balanced and inclusive approach to resource utilization. In Greece, the basic European and Greek national legislative framework that governs the management of animal (including fish) by-products is included in the Supplementary Materials.

In conclusion, the presented data offer a promising foundation for the development of a comprehensive plan for the exploitation of fishery discards and by-products in Greece. Strategic planning, sustainable practices, and stakeholder collaboration are crucial for realizing the full potential of these resources while safeguarding the marine ecosystem and promoting economic development.

## 4. Materials and Methods

### 4.1. Discards Data

National Fisheries Data Collection Framework Program (DCF-EPSAD) data were obtained from sampling on commercial vessels (seines and trawlers) in twelve regions of Greece (Figure 4) in the period 2013–2018. Specifically, this action of the National DCF includes recording the composition of commercial and discarded catches by observers on commercial vessels. Observers do not interact with the crew during fishing or stripping and record the number of individuals, total weight, and lengthwise composition by species after the process is over. Based on these measurements, discard ratios/fractions (discarded to commercial catch) are calculated, both for each species and overall, for a specific fishing gear and/or an area.

### 4.2. Landings Data

Estimates from the DCF-EPSAD regarding fishing productions (landings) for purse seiners and trawlers for the Greek seas (divided into twelve regions) were obtained in 2014. These estimates are based on a collection of information on (a) catches per vessel and (b) the fishing effort per vessel.

Data from catches that are not eventually sold at the 11 official Greek landing sites (see Table 2) were obtained from the official Central Markets and Fishery Organization (CMFO) records.

Data regarding fishing productions (landings) for purse seiners and trawlers, derived from the vessels’ Electronic Reporting System (OSPA, http://www.alieia.minagric.gr/ospa, accessed on 8 April 2019); these data were integrated in the HCMR’s fisheries database [58] and were exported for twelve areas for the years 2015–2017.Hellenic Statistical Authority ELSTAT’s [59] monthly landing data regarding the coastal fishing fleet (nets, longlines, traps) after 2015 (specifically 2016 and 2017 where available) were used to estimate coastal fisheries’ landings and discards. The reason was that, until the reference year 2015, ELSTAT’s Marine Fisheries Survey sampled motorized professional fishing vessels with a horsepower of 20 horsepower and above. From the reference year 2016 onwards, the survey covers a sample of all motorized professional fishing vessels, regardless of horsepower. However, it was not possible to carry out a spatial analysis as this information was not available from ELSTAT.

**Figure 5:**
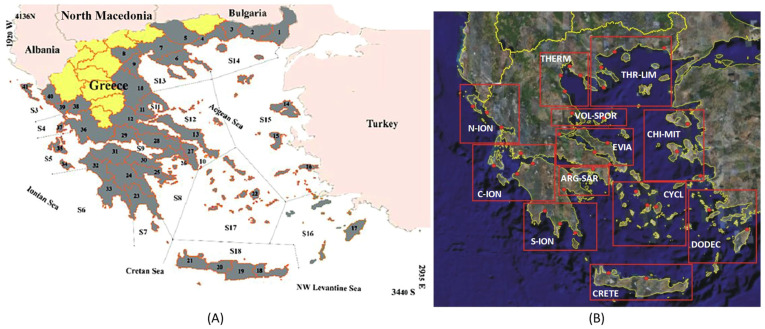
Areas for the data collection of the Hellenic Statistical Authority ELSTAT (**A**) and Areas for the Data of the National Fisheries Data Collection Framework Program EPSAD (**B**). Black numbers denote the Regional Units of Greece. The black S numbers on the left show the division to 16 fishing sub-areas (each enclosed by lines). Subareas 1 and 2 are outside Greek waters (Atlantic Ocean and North African Mediterranean coast, respectively). The EPSAD acronyms of the red squares (right) are explained in Table 7.

The matching of the CMFO fish landing sites to the DCF-EPSAD areas and their distribution in the three geographical sub-areas of the General Fisheries Commission for the Mediterranean GFCM (GSAs) is shown in Table 7 and Figure 5. The division of the Hellenic marine fisheries into 12 areas followed by the DCF-EPSAD largely corresponds to the division followed by the Hellenic Statistical Authority (ELSTAT). Specifically, ELSTAT divides the fishing activity in Greece into 16 regions (plus two overseas), (i.e., four additional regions compared to DCF) (Figure 5). The differences are found mainly in the Ionian Sea, where each region of the DCF-EPSAD corresponds to two areas of ELSTAT. In addition, South Evia is also a separate region in ELSTAT. The remaining areas correspond fully between EPSAD and ELSTAT.

To estimate the quantities of trawl and purse seine discards, the monthly landings of each of the 12 areas were multiplied by the ratio (R, discard ratio) of discards (D) to commercial (M) catches:

R = D/M (1)

Although data were available in most cases to calculate a discard ratio per month and per region, more than one estimate was made by applying different ratios (see below) in order to (a) account for the fact that, in some regions/seasons, the uncertainty was large due to a small sampling effort and (b) to perform an uncertainty analysis. In the cases (regions/seasons) where there were information gaps regarding the discard ratio, a general discard ratio was used, calculated either over the whole year for each region or at the level of the Aegean or Ionian seas for trawlers or purse seines. Listed below are the different approaches followed, of which the first three do not take into account the species caught but only the aggregated data per fishing gear, while the last one assesses at the species level and presents the aggregated results:

- Estimates based on universal discard ratios for each GSA (GSA 20 Ionian, GSA 22 Aegean, and GSA 23 Crete). A single discard ratio per gear was applied using all data collected per GSA for the entire time period, regardless of species.
- Estimates based on monthly discard ratios per GSA for the entire catch. For each gear, a different discard ratio was applied for each month based on data collected in a GSA that month, regardless of species.
- Estimates based on monthly discard ratios for the entire catch by DCF-EPSAD region. For each fishing gear, a different discard ratio was applied for each month and each DCF-EPSAD area based on the data collected in that area and that month, regardless of species.
- Estimates based on discard ratios by species for each GSA. For each species, a separate discard ratio was applied and estimated using all data for that species, by GSA and by fishing gear. The final estimate is obtained by summing up the discards of each species. However, in this particular analysis, to avoid details, the intermediate calculation steps are not listed.

Regarding the discards of artisanal fisheries, a single discard ratio was applied to ELSTAT’s landing data based on the study by Tzanatos et al. (2007) [60], where it was estimated at 10% of total captures (i.e., R = D/M = 0.11).

The third potential source of fishery biomass for the production of HAVBs is from FBP-3 from the processing of fishery and aquaculture products in the commercial and manufacturing chain. Many of the fishery and aquaculture products traded in supermarkets, street markets, and neighborhood fishmongers, as well as central markets where fish are sold, are returned to customers after being processed, i.e., the common “cleaning”. “Cleaning”, depending on the species of fish, may include gutting, scaling, cutting, skinning, filleting, removing gills, removing the head, etc. After this treatment, about 15–25% is removed (entrails, scales, backbones, and heads).

The estimation of this quantity is difficult to evaluate precisely. In Greece, the apparent consumption of fishery and aquaculture products (fish, mollusks, and crustaceans in fresh, frozen, or processed forms) presented in Table 8 takes into account all forms, even canned or smoked products, etc. To determine the FABs from the processing of fishery and aquaculture products in the commercial and processing chain, the following calculations have been made which are summarized in Table 8.

The data in Table 8 have been derived from ELSTAT (fisheries and aquaculture) and FAO (FIGIS—Fisheries Global Information System) (imports and exports) and the following assumptions:

- Regarding fishery production, only fish was considered. Cephalopods, bivalves, and crustaceans have been removed as they are not raw materials for HAVPs considered in this study (ELSTAT data).
- In the production of aquaculture, bivalves (mussels and oysters) and crustaceans have been removed as no raw materials to be used have been considered in this study (ELSTAT data).
- As the vast majority of imports concern frozen products, canned goods, and other processed products, only the ISSCAAP Division of diadromous fishes, freshwater fishes, and marine fishes were taken into account (data from FishstatJ of the FAO Global Fishery and Aquaculture Production Statistics).
- From exports, only the ISSCAAP Division of diadromous fishes, freshwater fishes, and marine fishes were taken into account (data from FishstatJ of the FAO Global Fishery and Aquaculture Production Statistics) [61].
- After these assumptions, an average apparent consumption of 70,026.71 tons (years 2017–2021) and a per capita consumption of 6.68 kg per inhabitant per year result. If the same percentages of fishery and aquaculture products are consumed throughout the Greek territory, then based on the apparent consumption, Table 8 will be obtained, after removing a conservative percentage of approximately 15% during the ‘cleaning’ process at retail points.

### 4.3. Collagen-Enriched Paste

The first step for the preparation of hydrolyzed collagen and acid-soluble collagen value chains was the production of collagen-enriched paste (CEP), which begins with the addition of 20% NaCl to the homogenized fish biomass, and the mechanical stirring of the mixture for 24 h to remove non-collagenous proteins. Filtration and removal of the filtrate followed, and the process was repeated three times until the aqueous phase was nearly colorless. The filtrate was removed using a 540-mesh permeable filter and the residue was rinsed twice with deionized water. Subsequently, dispersion of the residue took place in a solution of 0.5 M EDTA, pH 7.4 at a ratio of 1:5 (*w*/*w*), and mechanical stirring for 24 h to remove Ca^2+^ ions. Filtration and removal of the filtrate followed, and the process was repeated two more times. The filtrate was removed using a 540-mesh permeable filter and the residue was rinsed twice with deionized water. Finally, for the removal of lipid substances, the residue was dispersed in a solution of 10% iso-BuOH at a ratio of 1:10 (*w*/*v*) and subjected to mechanical stirring for 24 h. Filtration and removal of the filtrate followed, and the process was repeated two more times. The filtrate was removed using a 540-mesh permeable filter and the residue was rinsed twice with deionized water. All steps were performed at 8 °C. After the completion of the procedure, CEP was obtained.

### 4.4. Hydrolyzed Collagen/Collagen Peptides

For the isolation of hydrolyzed collagen, CEP was suspended in a solution of 10 mM HCl in a ratio of 1:10 (*w*/*v*). The mixture was heated for 48 h at 60 °C [62]. The hydrolyzed collagen was separated after removing insoluble components by filtration using a 210-mesh permeable filter and then lyophilized.

### 4.5. Acid-Soluble Collagen (ASC)

CEP was suspended in a solution of 0.5 M acetic acid in a ratio of 1:10 (*w*/*v*). The mixture was then mechanically stirred for 24 h at 8 °C. After filtration and removal of the filtrate, the process was repeated two more times. After centrifugation at 6000× *g* for 30 min, the supernatant was collected and NaCl was added to a final concentration of 0.9 M to precipitate the acid-soluble collagen (ACS). The solution was left at rest at 4 °C overnight. The ASC was collected as a pellet after centrifugation at 10,000× *g* for 1 h. The pellet was redissolved in an appropriate volume of 0.5 M acetic acid and subjected to dialysis, initially against 0.1 M acetic acid and then against deionized water. After lyophilization, high-purity ACS was obtained.

### 4.6. Fish Meal—Fish Oil Potential Yield Estimation

The industrial mass balance for a prospective manufacturer consists of three major fractions of the raw material: solids (fat-free dry matter), oil, and water. The actual composition of the raw material will vary, particularly that of the oil content; therefore, the estimates were calculated based on analytical results along with a literature review of industrial best practices. Thus, the estimates are sufficient to illustrate the general trend.

To assess the nutritional value and the potential yields of Mediterranean fish species, samples of bogue (*Boops boops*), European pilchard (*Sardina pilchardus*), European anchovy (*Engraulis encrasicolus*), mackerel (*Scomber scombrus*), common pandora (*Pagellus erythrinus*), large-eye dentex (*Dentex macrophthalmus*), common scad (*Trachurus trachurus*), white seabream (*Diplodus sargus*), and blotched picarel (*Spicara maena*) were homogenized, lyophilized, and analyzed for dry matter and ash according to AOAC (2005). Moisture content was measured after drying the samples at 105 °C for 24 h, ash was determined after ignition at 500 °C for 12 h, crude protein content was analyzed by using the Kjeldahl method (N × 6.25) (Kjeltec 8100, FOSS, Denmark), and total fat was estimated gravimetrically by using SoxtecTM (FOSS, 2050 automated analyzer, Denmark) and petroleum ether extraction. Additionally, to receive accurate data for the yield estimations of the discarded Mediterranean fish species, small-spotted catshark (*Scyliorhinus canicular*), shortnose greeneye (*Chlorophthalmus agassizi*), velvet belly lanternshark (*Etmopterus spinax*), blackmouth catshark (*Galeus melastomus*), four-spot megrim (*Lepidorhombus bosccii*), hollowsnout grenadier (*Coelorhynchus coelorhynchus*), greater fork-beard (*Phycis blennoides*), silver scabbardfish (*Lepidopus caudatus*), agrentine (*Argentina sphyraena*), boarfish (*Capros aper*), blackbelly rosefish (*Helicolemus dactylopterus*), silvery pout (*Gadiculus argenteus*), African armored searobin (*Peristedion cataphractum*), Phaeton dragonet (*Synchiropus phaeton*), blackbellied angler (*Lophius budegassa*), and silver roughy (*Hoplostethus mediterraneus*) proximate compositions were analyzed.

### 4.7. Fish Silage Separation Yields

To accurately assess the potential of ensilaging as a valorization method, the experimental ensilaging protocol was applied to samples of bogue (*B. boops*), European pilchard (*S. pilchardus*), European anchovy (*E. encrasicolus*), and a mixture of the three in equal parts. Three samplings (15, 30, and 80 days) followed to examine the stability of the silage as well as the effects of long-term storage on the nutritional profile. Afterwards, to investigate an indicative scenario in commercial conditions, according to the received quantities, a mixture was formed containing 40.2% bogue (*B. boops*), 10% mackerel (*S. scombrus*), 10.1% common pandora (*P. erythrinus*), 8.9% large-eye dentex (*D. macrophthalmus*), 10% common scad (*T. trachurus*), 10.7% white seabream (*D. sargus*), and 10.1% blotched picarel (*S. maena*).

For the preparation of the fish silages, 1.5 kg of homogenized fish mince was carefully weighted in glass vessels with a 2 L volume. Afterwards, 99% formic acid (Analytical reagent grade, Carlo Erba, Cornaredo, Italy) was added with simultaneous mixing, while the pH was constantly monitored with a Seven excellence Multiparameter pH meter (Melter Toledo, Singapore) until the pH value reached the target of 3.5 to 4 to prevent bacterial growth. Approximately, 20 mL of formic acid for 1 kg of fish mince are necessary to achieve the appropriate conditions for liquefaction, which was completed after one day. The fish silage could be either utilized in its crude liquid form or be subjugated to freeze drying to produce a protein concentrate.

### 4.8. Marine Mineral Trace Elements and Protein Complexes

Samples from the experimental fish silages were removed and placed in 50 mL falcon tubes, which were then weighed to estimate the yields gravimetrically. The tubes were then centrifuged at 3000 rpm with a temperature of 10 °C for 10 min in a refrigerated centrifuge. Afterwards, the sample was separated into three phases: lipid, hydrolyzed protein, and sludge. The upper two layers were then removed, and lipid extraction was performed in the remaining phase to isolate the marine trace elements and protein complexes using petroleum ether. Each extraction cycle consisted of the addition of 20 mL of petroleum ether in the falcon tubes, vigorous mixing in the vortex, centrifugation at 3000 rpm for 10 min, and isolation of the upper phase (organic solvent). The cycles were repeated until the solvent appeared colorless. The tubes were then placed in a −20 °C freezer overnight and lyophilized to remove the remaining moisture from the final product, which was analyzed for proximate composition, phosphorus (P), calcium (Ca), magnesium (Mg), manganese (Mn), iron (Fe), and zinc (Zn) using Microwave Plasma-Atomic Emission Spectroscopy (MP-AES).

### 4.9. Marine Hydrolyzed Protein Dietary Supplements

The two phases from the previous procedure (lipid and hydrolyzed protein layers) were placed in falcon tubes and an extraction protocol using petroleum ether was performed to separate the phases, each extraction cycle consisting of the addition of 20 mL of petroleum ether in the falcon tubes, vigorous mixing in the vortex, centrifugation at 3000 rpm for 10 min, and isolation of the upper phase (organic solvent). The cycles were repeated until the solvent appeared colorless. The tubes were then placed in a −20 °C freezer overnight and lyophilized to remove the remaining moisture from the final product which was analyzed for proximate composition and amino acid profile after acid hydrolysis (6 N HCl, 110 °C, 24 h) and derivatization by AccQ-Tag™ Ultra according to Kotzamanis et al. (2020) [63]. DL-Norvaline (Sigma-Aldrich, Darmstadt, Germany) 2.5 mM was used as an internal standard. UPLC was performed on an Acquity system (Waters Corporation, Milford, MA, USA) equipped with a PDA detector, and the detection wavelength was set at 260 nm. The column used was a BEH C18 column (100 mm × 2.1 mm i.d., 1.7 μm) from Waters. The flow rate was 0.7 mL/min and the column temperature was kept at 55 °C. Peak identification and integration were performed by the software Empower v.2.0 (Waters Corporation, Milford, MA, USA) using Amino Acid Standard H (Thermo Scientific Waltham, MA USA) as an external standard.

### 4.10. Omega-3 Fatty Acid Supplements

The isolated lipid phase was placed in pre-weighed evaporation vessels to calculate the yield gravimetrically. The vessels were then placed on a rotary evaporator using a water bath that was set at 40 °C. The vessels were carefully dried, placed on a desiccator to remove excess moisture, and weighed on 4-digit analytical balance to assess the lipid content. Additionally, fatty acid methyl-esters (FAMEs) were analyzed using an Agilent GC-7890 B gas chromatograph (Agilent Technologies, Santa Clara, CA, USA) equipped with a flame-ionization detector (GC-FID) and a DB-23 capillary column (60 m × 0.25 mm i.d. × 0.15 µm film thickness) (Agilent, Santa Clara, CA, USA). Helium was used as carrier gas at 2 mL/min constant flow; the split ratio was 1:50, and the injected volume was 1.0 μL. The thermal gradient was 50 °C for 1 min, 50 °C to 175 °C at 25 °C/min, 175 °C to 230 °C at 4 °C/min, and held at 230 °C for 15 min. The injector and detector temperatures were maintained at 250 and 280 °C, respectively. Fatty acids were identified by comparison with a known standard mixture (Supelco 37 Component FAMΕ Mix).

## 5. Conclusions

This is the first attempt to systematically record and analyze the available quantities of FBPD in Greece in a spatial and temporal way in an effort to create a national exploitation Master Plan for the valorization of this unavoidable and unwanted biomass. The quantities are not insignificant and can justify the creation of a pilot plant that can produce FBPD for both human consumption and as ingredients for animal feeds. If the quantities from the continuously growing share of filleting products from the Greek aquaculture of European seabass, gilthead seabream, red seabream (*P. major*), and meagre will be added to these quantities, there is no doubt that in the near future, the establishment of pilot plants in Greece for the valorization of this biomass will be possible. The Maritime, Fisheries, and Aquaculture Fund (EMFAF) may fund initiatives, where fisheries also participate in an effort to enhance the circular economy and support innovative projects that contribute to the sustainable exploitation and management of aquatic and maritime resources.

## Figures and Tables

**Table 1:** High-added-value biomolecules (HAVBs) that can be produced from the utilization of fishery by-products and discarded fish (FBPD).

**1. HAVB from fresh or frozen FBPD** A1. Acid-Soluble Collagen (ASC) A2. Hydrolyzed collagen/collagen peptides B1. Fish meal B2. Fish oil C1. Marine hydrolyzed protein dietary supplements (food grade) C2. Marine mineral trace elements and protein complexes (food grade)
**2. HAVB from ensilaged FBPD** D1. Omega-3 fatty acid supplements (for animal feed) D2. Marine hydrolyzed protein dietary supplements (for animal feed) D3. Marine mineral trace elements and protein complexes (for animal feed) E1. Fish silage protein concentrate (for animal feed)

**Table 2:** Fish catch that is not sold at the Greek fish landing sites and may be valorized for the production of HAVBs; quantities per year and area. Quantities are given in tonnes.

Area—GSA	Area Code	Area National Fisheries Data Col/on Program	CMFO Fish Landing Site	Total
Ionian Sea—20	N-ION	North Ionian Sea	Preveza	1.94
	C-ION	Central Ionian Sea	Patras, Messolonghi	27.62
	S-ION	South Ionian Sea	-	
Aegean Sea—22	THR-LIM	Thrace and Lemnos	Kavala, Alexandroupolis	114.42
	THERM	Thermaikos	Thessaloniki	272.66
	VOL-SPOR	Volos and Sporades	Volos	-
	CHI-MIT	Chios and Lesbos	Chios	3.39
	EVIA	Evia	Chalkida	-
	ARGSAR	Argosaronikos	Piraeus	244.64
	CYCL	Cyclades	-	41.28
	DODEC	Dodecanese	Kalymnos	0.14
Crete—23	CRETE	Crete	Chania	16.05
Grand total				722.14

**Table 3:** Discarded catches from trawlers and purse seiners (average values for the period 2014–2017) in Greece that may be valorized for the production of HAVBs; quantities per year and area. Quantities are given in tonnes.

Area—GSA	Area Code	Area National Fisheries Data Collection Program	CMFO Fish Landing Site	Total
Ionian Sea—20	N-ION	North Ionian Sea	Preveza	255.3
	C-ION	Central Ionian Sea	Patras, Messolonghi	523.0
	S-ION	South Ionian Sea	-	21.3
Aegean Sea—22	THR-LIM	Thrace and Lemnos	Kavala, Alexandroupolis	3069.7
	THERM	Thermaikos	Thessaloniki	992.2
	VOL-SPOR	Volos and Sporades	Volos	266.9
	CHI-MIT	Chios and Lesbos	Chios	613.9
	EVIA	Evia	Chalkida	347.1
	ARGSAR	Argosaronikos	Piraeus	457.8
	CYCL	Cyclades	-	745.8
	DODEC	Dodecanese	Kalymnos	149.9
Crete—23	CRETE	Crete	Chania	210.4
Grand total				7653.3

**Table 4:** Category 3 fish by-products (FBP-3) from fish processing in the commercial and retail processing chain (average values for the period 2017–2021) in Greece that may be valorized for the production of HAVBs; quantities per year and area. Quantities are given in tonnes.

Area—GSA	Area Code	Area National Fisheries Data Collection Program	CMFO Fish Landing Site	Total
Ionian Sea—20	N-ION	North Ionian Sea	Preveza	320.59
	C-ION	Central Ionian Sea	Patras. Messolonghi	649.44
	S-ION	South Ionian Sea	-	204.92
Aegean Sea—22	THR-LIM	Thrace and Lemnos	Kavala. Alexandroupolis	563.26
	THERM	Thermaikos	Thessaloniki	2055.88
	VOL-SPOR	Volos and Sporades	Volos	689.55
	CHI-MIT	Chios and Lesbos	Chios	195.31
	EVIA	Evia	Chalkida	509.21
	ARGSAR	Argosaronikos	Piraeus	3821.26
	CYCL	Cyclades	-	
	DODEC	Dodecanese	Kalymnos	328.44
Crete—23	CRETE	Crete	Chania	625.59
		Peloponnese		540.55
Grand total				10,504.01

**Table 5:** Total potential biomass sources in Greece for the production of HAVBs from FBPD per year and area from the three categories (fish catch that is not sold at the Greek fish landing sites, discarded catches from trawlers and purse seines, and from fish processing in the commercial and retail processing chain). Quantities are given in tonnes.

Area—GSA	Area Code	Area National Fisheries Data Collection Program	CMFO Fish Landing Site	Total
Ionian Sea—22	N-ION	North Ionian Sea	Preveza	577.71
	C-ION	Central Ionian Sea	Patras, Messolonghi	1200.07
	S-ION	South Ionian Sea	-	226.32
Aegean Sea—22	THR-LIM	Thrace and Lemnos	Kavala, Alexandroupolis	3747.49
	THERM	Thermaikos	Thessaloniki	3320.75
	VOL-SPOR	Volos and Sporades	-	956.35
	CHI-MIT	Chios and Lesbos	Chios	812.49
	EVIA	Evia	Chalkida	856.41
	ARGSAR	Argosaronikos	Piraeus	4523.82
	CYCL	Cyclades	-	787.09
	DODEC	Dodecanese	Kalymnos	478.39
Crete—23	CRETE	Crete	Chania	851.93
		Peloponnese		540.55
Grand total				18,879.38

**Table 6:** Yields (in % of initial fresh weight) estimated by the current study for the production of high-added-value biomolecules of (HAVBs) that can be produced from the utilization of fishery by-products and discarded fish (FBPD). Categories A and C are intended for human consumption and use while categories B, D, and E are destined for animal feed ingredients.

List of Potential Products	Yield (%)
A1. Acid-Soluble Collagen (ASC)	5.37
A2. Hydrolyzed collagen/collagen peptides	6.61
B1. Fish meal	21.85
B2. Fish oil	2.01
C1. Marine hydrolyzed protein dietary supplements (food grade)	11.04
C2. Marine mineral trace elements and protein complexes (food grade)	15.18
D1. Omega-3 fatty acid supplements (for animal feed)	2.26
D2. Marine hydrolyzed protein dietary supplements (for animal feed)	11.04
D3. Marine mineral trace elements and protein complexes (for animal feed)	15.18
E1. Fish silage protein concentrate (for animal feed)	20.72

**Table 7:** Correlation between GSA areas, data collection Framework (EPSAD) areas, and CMFO fish landing sites.

Area—GSA	DCF—EPSAD		CMFO Fish Landing Sites
Ionian Sea—20	N-ION	North Ionian Sea	Preveza
	C-ION	Central Ionian Sea	Patras, Messolonghi
	S-ION	South Ionian Sea	-
Aegean Sea—22	THR-LIM	Thrace and Lemnos	Kavala, Alexandroupolis
	THERM	Thermaikos	Thessaloniki
	VOL-SPOR	Volos and Sporades	Volos
	CHI-MIT	Chios and Lesbos	Chios
	EVIA	Evia	Chalkida
	ARGSAR	Argosaronikos	Piraeus
	CYCL	Cyclades	-
	DODEC	Dodecanese	Kalimnos
Crete—23	CRETE	Crete	Chania

**Table 8:** Calculation of apparent consumption of fresh or frozen fish only for determination of category 3 fish by-products (FBP-3) from the processing of fishery and aquaculture products in the commercial and processing chain in Greece.

Year	Fisheries (Tonnes)	Aquaculture (Tonnes)	Imports (Tonnes)	Exports (Tonnes)	Apparent Consumption (Tonnes)	Per Capita Consumption (kg)
2017	62,347.10	106,230.00	19,281.00	120,891.00	66,967.10	6.39
2018	62,065.20	110,239.60	26,259.00	122,421.00	76,142.80	7.26
2019	67,107.80	104,944.00	31,624.67	125,779.40	77,897.06	7.43
2020	55,884.90	112,914.70	27,709.47	132,542.40	63,966.63	6.10
2021	45,833.20	130,062.40	28,521.14	139,256.80	65,159.94	6.22

## Data Availability

The data that support the findings of this study are available from the corresponding authors upon request.

## Supplementary Materials

The following supporting information can be downloaded at: https://www.mdpi.com/article/10.3390/md22060264/s1, Table S1. Fish catch that is not sold at the Greek fish landing sites and can be valorized to produce High Added Value Biomolecules (HAVB); quantities per month (1-12) and area. Quantities are given in tonnes; Table S2. Discarded catches from trawlers and purse seiners in Greece that can be valorized to produce High Added Value Biomolecules (HAVB); quantities per month (1-12) and area. Quantities are given in tonnes; Table S3. Category 3 fish by-products (FBP-3) from fish processing in the commercial and retail processing chain in Greece that can be valorized to produce High Added Value Biomolecules (HAVB); quantities per month (1-12) and area. Quantities are given in tonnes; Table S4. Total potential biomass sources in Greece to produce High Added Value Biomolecules (HAVB) from fishery by-products and discarded fish (FBPD) per year and area from the three categories (fish catch that is not sold at the Greek fish landing sites, discarded catches from trawlers and purse seines and fish processing in the commercial and retail processing chain. Quantities are given in tonnes; Table S5. Proximate Composition of Mediterranean fish species. Table S6. Comparison of Mediterranean fish species proximate composition before and after 15, 30 and 80 days of ensilaging; Table S7. Proximate composition of hydrolyzed protein and sludge powder from ensilaged Mediterranean unsold fish species. Table S8. Proximate composition of discarded Mediterranean fish species; Table S9. Comparison of bogue (Boops boops) fatty acid profile before and after 15 and 80 days of ensilaging; Table S10. Comparison of European anchovy (Engraulis encrasicolus) fatty acid profile before and after 15 and 80 days of ensilaging; Table S11. Comparison of European pilchard (Sardina pilchardus) fatty acid profile before and after 15 and 80 days of ensilaging; Table S12. Comparison of the mix of bogue (Boops boops), European anchovy (Engraulis encrasicolus), and European pilchard (Sardina pilchardus) fatty acid profile before and after 15 and 80 days of ensilaging; Table S13. Comparison of the mix of Mediterranean unsold fish species fatty acid profile before and after 80 days of ensilaging; Table S14. Comparison of bogue (Boops boops) mineral profile before and after 80 days of ensilaging; Table S15. Comparison of European anchovy (Engraulis encrasicolus) mineral profile before and after 80 days of ensilaging; Table S16. Comparison of European pilchard (Sardina pilchardus) mineral profile before and after 80 days of ensilaging; Table S17. Comparison of the mix of bogue (Boops boops), European anchovy (Engraulis encrasicolus) and European pilchard (Sardina pilchardus) mineral profile before and after 80 days of ensilaging; Table S18. Comparison of the mix of Mediterranean unsold fish species mineral profile before and after 80 days of ensilaging; Table S19. Mediterranean unsold fish species amino acid profile [64,65,66].
