# Supplementary material for: Status of Fishery Discards and By-Products in Greece and Potential Valorization Scenarios towards a National Exploitation Master Plan
Source: Mar Drugs. 2024 Jun 7;22(6):264. doi: 10.3390/md22060264 (PMC11205227; doi:10.3390/md22060264)
Supplement: Supplementary file 1 [file marinedrugs-22-00264-s001.zip › marinedrugs-3034618-supplementary.pdf]

Table S1. Fish catch that is not sold at the Greek fish landing sites and can be valorized to produce High Added Value Biomolecules (HAVB); quantities per month (1-12) and area. Quantities are given in tonnes.

	Area code	Area National Fisheries Data Col/on Program	CMFO fish landing site	1	2	3	4	5	6	7	8	9	10	11	12	Total
	N-ION	North Ionian Sea	Preveza	0.12	0.12	0.2	0.25	0.22	0.1	0.13	0.14	0.12	0.23	0.16	0.13	**1.94**
**Ionian Sea - 20**	C-ION	Central Ionian Sea	Patras, Messolonghi	1.76	1.66	2.88	3.62	3.15	1.39	1.89	2.05	1.77	3.35	2.32	1.79	**27.62**
	S-ION	South Ionian Sea	-													
	THR-LIM	Thrace and Lemnos	Kavala, Alexandroupolis	7.28	6.88	11.92	15	13.04	5.78	7.84	8.5	7.32	13.86	9.61	7.4	**114.42**
	THERM	Thermaikos	Thessaloniki	17.34	16.39	28.4	35.75	31.08	13.77	18.69	20.25	17.44	33.02	22.9	17.64	**272.66**
	VOL-SPOR	Volos and Sporades	Volos													
	CHI-MIT	Chios and Lesbos	Chios	0.22	0.2	0.35	0.44	0.39	0.17	0.23	0.25	0.22	0.41	0.28	0.22	**3.39**
**Aegean Sea - 22**	EVIA	Evia	Chalkida													
	ARGSAR	Argosaronikos	Piraeus	15.56	14.7	25.48	32.08	27.89	12.35	16.77	18.17	15.65	29.63	20.55	15.83	**244.64**
	CYCL	Cyclades	-	2.63	2.48	4.3	5.41	4.71	2.08	2.83	3.07	2.64	5	3.47	2.67	**41.28**
	DODEC	Dodecanese	Kalymnos	0.01	0.01	0.01	0.02	0.02	0.01	0.01	0.01	0.01	0.02	0.01	0.01	**0.14**
**Crete - 23**	CRETE	Crete	Chania	1.02	0.96	1.67	2.1	1.83	0.81	1.1	1.19	1.03	1.94	1.35	1.04	**16.05**
**Grand total**				45.94	43.4	75.21	94.67	82.33	36.46	49.49	53.63	46.2	87.46	60.65	46.73	**722.14**

Table S2. Discarded catches from trawlers and purse seiners in Greece that can be valorized to produce High Added Value Biomolecules (HAVB); quantities per month (1-12) and area. Quantities are given in tonnes.

Area - GSA	Area code	Area National Fisheries Data Col/on Program	CMFO fish landing sites	1	2	3	4	5	6	7	8	9	10	11	12	Total
Ionian Sea - 20	N-ION	North Ionian Sea	Preveza	8.1	5.6	11.6	31.3	22.2	10.3	19.2	29.8	29.6	40.1	36.8	10.6	**255.3**
	C-ION	Central Ionian Sea	Patras, Messolonghi	64.2	51.5	39.9	36.7	32.5	17.6	20.2	32.6	38.2	65.8	58.4	65.4	**523.00**
	S-ION	South Ionian Sea	-	2.6	1.9	2.9	2.8	1.6	0.5	0.4	1.1	0.2	2.1	4	1.3	**21.3**
Aegean Sea - 22	THR-LIM	Thrace and Lemnos	Kavala, Alexandroupolis	282.9	258.6	251.4	211.9	221.6	439.9	159.7	246.5	142.5	329	285.5	240.3	**3069.7**
	THERM	Thermaikos	Thessaloniki	86.8	95.1	108.1	89	86.6	49.6	7.6	20.8	8.2	185.4	141.5	113.5	**992.2**
	VOL-SPOR	Volos and Sporades	Volos	17.4	16.6	29	29.4	34.5	34.7	4.9	9	3.4	43.8	24.5	19.6	**266.9**
	CHI-MIT	Chios and Lesbos	Chios	37.6	46.6	64.8	62.7	68.4	20.9	44.2	62.2	48.1	70	55.1	33.2	**613.9**
	EVIA	Evia	Chalkida	36.2	22	39	27.2	31	37	12.3	25.2	9.3	38.1	46.4	23.5	**347.1**
	ARGSAR	Argosaronikos	Piraeus	36.6	36.6	46.4	28.4	28.2	108.1	7.4	21.4	6.3	33	62.6	42.9	**457.8**
	CYCL	Cyclades	-	56	64.5	95.2	94.7	106.6	20.6	9.2	14.2	18	126.4	93.6	46.8	**745.8**
	DODEC	Dodecanese	Kalymnos	16.4	17.3	22.5	17.9	14.6	2.7	1	2.2	4.3	19.6	17.9	13.4	**149.9**
**Crete - 23**	CRETE	Crete	Chania	19.5	25.5	29	22.3	16.1	3.8	1.5	3.1	1.3	41.2	25.6	21.4	**210.4**
**Grand total**				664.3	641.8	739.8	654.3	663.9	745.7	287.6	468.2	309.4	994.6	851.8	631.9	**7653.3**

Table S3. Category 3 fish by-products (FBP-3) from fish processing in the commercial and retail processing chain in Greece that can be valorized to produce High Added Value Biomolecules (HAVB); quantities per month (1-12) and area. Quantities are given in tonnes.

**Area - GSA**	**Area code**	**Area National Fisheries Data Col/on Program**	**CMFO fish landing sites**	**1**	**2**	**3**	**4**	**5**	**6**	**7**	**8**	**9**	**10**	**11**	**12**	**Total**
**Ionian Sea - 20**	N-ION	North Ionian Sea	Preveza	30.06	30.06	27.55	27.55	25.05	22.54	22.54	20.04	27.55	30.06	30.06	27.55	**320.59**
	C-ION	Central Ionian Sea	Patras. Messolonghi	60.89	60.89	55.81	55.81	50.74	45.66	45.66	40.59	55.81	60.89	60.89	55.81	**649.44**
	S-ION	South Ionian Sea	-	19.21	19.21	17.61	17.61	16.01	14.41	14.41	12.81	17.61	19.21	19.21	17.61	**204.92**
**Aegean Sea - 22**	THR-LIM	Thrace and Lemnos	Kavala. Alexandroupolis	52.81	52.81	48.41	48.41	44	39.6	39.6	35.2	48.41	52.81	52.81	48.41	**563.26**
	THERM	Thermaikos	Thessaloniki	192.74	192.74	176.68	176.68	160.62	144.55	144.55	128.49	176.68	192.74	192.74	176.68	**2055.88**
	VOL-SPOR	Volos and Sporades	Volos	64.65	64.65	59.26	59.26	53.87	48.48	48.48	43.1	59.26	64.65	64.65	59.26	**689.55**
	CHI-MIT	Chios and Lesbos	Chios	18.31	18.31	16.78	16.78	15.26	13.73	13.73	12.21	16.78	18.31	18.31	16.78	**195.31**
	EVIA	Evia	Chalkida	47.74	47.74	43.76	43.76	39.78	35.8	35.8	31.83	43.76	47.74	47.74	43.76	**509.21**
	ARGSAR	Argosaronikos	Piraeus	358.24	358.24	328.39	328.39	298.54	268.68	268.68	238.83	328.39	358.24	358.24	328.39	**3821.26**
	CYCL	Cyclades	-													
	DODEC	Dodecanese	Kalymnos	30.79	30.79	28.23	28.23	25.66	23.09	23.09	20.53	28.23	30.79	30.79	28.23	**328.44**
**Crete - 23**	CRETE	Crete	Chania	58.65	58.65	53.76	53.76	48.87	43.99	43.99	39.1	53.76	58.65	58.65	53.76	**625.59**
		Peloponnese		50.68	50.68	46.45	46.45	42.23	38.01	38.01	33.78	46.45	50.68	50.68	46.45	**540.55**
**Grand total**				**984.75**	**984.75**	**902.69**	**902.69**	**820.63**	**738.56**	**738.56**	**656.50**	**902.69**	**984.75**	**984.75**	**902.69**	**10,504.01**

Table S4. Total potential biomass sources in Greece to produce High Added Value Biomolecules (HAVB) from fishery by-products and discarded fish (FBPD) per year and area from the three categories (fish catch that is not sold at the Greek fish landing sites, discarded catches from trawlers and purse seines and fish processing in the commercial and retail processing chain. Quantities are given in tonnes.

**Area - GSA**	**Area code**	**Area National Fisheries Data Col/on Program**	**CMFO fish landing sites**	**1**	**2**	**3**	**4**	**5**	**6**	**7**	**8**	**9**	**10**	**11**	**12**	**Total**
**Ionian Sea - 22**	N-ION	North Ionian Sea	Preveza	38.28	35.78	39.35	59.1	47.47	32.94	41.87	49.98	57.27	70.39	67.02	38.28	**577,71**
	C-ION	Central Ionian Sea	Patras, Messolonghi	126.85	114.05	98.59	96.13	86.39	64.65	67.75	75.24	95.78	130.04	121.61	123	**1,200.07**
	S-ION	South Ionian Sea	-	21.81	21.11	20.51	20.41	17.61	14.91	14.81	13.91	17.81	21.31	23.21	18.91	**226,32**
**Aegean Sea - 22**	THR-LIM	Thrace and Lemnos	Kavala, Alexandroupolis	342.99	318.29	311.73	275.31	278.64	485.28	207.14	290.2	198.23	395.67	347.92	296.11	**3,747,49**
	THERM	Thermaikos	Thessaloniki	296.88	304.23	313.18	301.43	278.3	207.92	170.84	169.54	202.32	411.16	357.14	307.82	**3,320,75**
	VOL-SPOR	Volos and Sporades	-	82.05	81.25	88.26	88.66	88.37	83.18	53.38	52.1	62.66	108.45	89.15	78.86	**956,35**
	CHI-MIT	Chios and Lesbos	Chios	56.13	65.11	81.93	79.92	84.05	34.8	58.16	74.66	65.1	88.72	73.69	50.2	**812,49**
	EVIA	Evia	Chalkida	83.94	69.74	82.76	70.96	70.78	72.8	48.1	57.03	53.06	85.84	94.14	67.26	**856,41**
	ARGSAR	Argosaronikos	Piraeus	410.4	409.54	400.27	388.87	354.63	389.13	292.85	278.4	350.34	420.87	441.39	387.12	**4,523,82**
	CYCL	Cyclades	-	58.63	66.98	99.5	100.11	111.31	22.68	12.03	17.27	20.64	131.4	97.07	49.47	**787,09**
	DODEC	Dodecanese	Kalymnos	47.2	48.1	50.74	46.15	40.28	25.8	24.1	22.74	32.54	50.41	48.7	41.64	**478,39**
**Crete - 23**	CRETE	Crete	Chania	79.17	85.11	84.43	78.16	66.8	48.6	46.59	43.39	56.09	101.79	85.6	76.2	**851,93**
		Peloponnese		50,68	50.68	46.45	46.45	42.23	38.01	38.01	33.78	46.45	50.68	50.68	46.45	**540.55**
**Grand total**				**1,694.99**	**1,669.95**	**1,717.70**	**1,651.66**	**1,566.86**	**1,520.72**	**1,075.65**	**1,178.23**	**1,258.29**	**2,066.71**	**1,897.30**	**1,581.32**	**18,879.38**

Table S5. Proximate Composition of Mediterranean fish species.

**Sample**	***B. boops***	***T. trachurus***	***S. scombrus***	***D. macrophthalmus***	***P. erythrinus***	***D. sargus***
**Crude Protein (%)**	16.49	17.73	17.41	17.90	16.91	15.87
**Crude fat (%)**	4.91	3.30	1.19	1.91	1.75	1.17
**Ash (%)**	3.47	3.13	2.97	6.29	5.36	4.55
**Moisture (%)**	74.68	75.37	77.71	74.31	75.70	77.31

Table S6. Comparison of Mediterranean fish species proximate composition before and after 15, 30 and 80 days of ensilaging.

**Sample**	***B. boops***	***B. Boops* 15D**	***B. Boops* 30D**	***B. Boops* 80D**
**Crude Protein (%)**	16.63	16.85	16.40	17.42
**Crude fat (%)**	2.01	1.93	1.44	1.26
**Ash (%)**	4.16	3.50	3.71	3.55
**Moisture (%)**	76.94	76.41	75.58	75.63
**Sample**	***E. encrasicolus***	***E. encrasicolus* 15D**	***E. encrasicolus* 30D**	***E. encrasicolus* 80D**
**Crude Protein (%)**	17.96	18.49	17.99	18.37
**Crude fat (%)**	4.44	3.96	3.02	1.87
**Ash (%)**	2.90	2.70	2.67	2.73
**Moisture (%)**	74.28	72.37	72.63	73.28
**Sample**	***S. pilchardus***	***S. pilchardus* 15D**	***S. pilchardus* 30D**	***S. pilchardus* 80D**
**Crude Protein (%)**	16.18	15.95	15.88	16.23
**Crude fat (%)**	5.81	6.05	4.48	2.80
**Ash (%)**	3.34	3.27	3.31	3.21
**Moisture (%)**	74.05	72.68	72.33	73.19
**Sample**	**Mix HCMR**	**Mix HCMR 15D**	**Mix HCMR 30D**	**Mix HCMR 80D**
**Crude Protein (%)**	17.24	16.69	16.08	16.69
**Crude fat (%)**	4.07	3.96	2.48	2.16
**Ash (%)**	3.31	3.23	3.02	3.10
**Moisture (%)**	74.86	74.26	74.74	74.26
**Sample**	**Mix Discards**	**Mix Discards 20D**	**Mix Discards 80D**	
**Crude Protein (%)**	17.22	19.18	18.16	
**Crude fat (%)**	2.72	2.96	2.26	
**Ash (%)**	3.98	3.76	4.16	
**Moisture (%)**	75.54	72.50	72.87	

Table S7. Proximate composition of hydrolyzed protein and sludge powder from ensilaged Mediterranean unsold fish species.

Sample	Fish hydrolyzed protein powder	Sludge powder
**Crude Protein (%)**	72.83	56.58
**Crude fat (%)**	-	3.26
**Ash (%)**	10.01	23.03
**Moisture (%)**	15.07	8.04

Table S8. Proximate composition of discarded Mediterranean fish species.

Sample	*Mediterranean fish discards*
**Crude Protein (%)**	18.38
**Crude fat (%)**	1.87
**Ash (%)**	4.58
**Moisture (%)**	76.61

Table S9. Comparison of bogue (*Boops boops)* fatty acid profile before and after 15 and 80 days of ensilaging.

FA	*B. boops*	*B. boops 15D*	*B. boops 80D*
**C14:0** (Myristic Acid)	5.49	8.05	8.16
**C16:0** (Palmitic Acid)	18.24	28.11	28.48
**C16:1 n7** (Palmitoleic Acid)	6.18	8.40	8.51
**C18:0** (Stearic Acid)	5.60	8.24	8.37
**C18:1 n9 cis** (Oleic Acid)	8.31	10.26	10.36
**C18:1 n7** (Vaccenic acid)	2.63	3.78	3.82
**C18:2 n-6 cis** (Linoleic Acid)	1.44	1.21	1.10
**C18:4 n3** (Stearidonic acid)	1.37	0.37	0.30
**C20:1 n9** (Gondoic Acid)	1.76	2.12	2.27
**C20:3 n3** (Dihomo-α-linolenic Acid)	1.95	0.86	0.64
**C20:5 n-3** (Eicosapentaenoic acid EPA)	6.69	2.10	1.57
**C22:6 n-3** (Docosahexaenoic Acid DHA)	17.56	5.59	4.12
**Saturated Fatty Acids**	32.13	48.35	48.88
**Monounsaturated Fatty acids**	24.04	30.24	30.88
**Polyunsaturated Fatty acids**	31.46	11.63	9.16
**Σ n-3**	28.92	9.42	7.11
**Σ n-6**	2.54	2.22	2.04
**Σ n-3/Σ n-6**	11.38	4.25	3.48
**EPA+DHA**	24.25	7.69	5.69
**EPA/DHA**	0.38	0.38	0.38
**DHA/EPA**	2.62	2.67	2.62

Table S10. Comparison of European anchovy (*Engraulis encrasicolus)* fatty acid profile before and after 15 and 80 days of ensilaging.

FA	*E.encrasicolus*	*E.encrasicolus 15D*	*E.encrasicolus 80D*
**C14:0** (Myristic Acid)	7.16	9.48	10.08
**C16:0** (Palmitic Acid)	20.73	28.17	30.00
**C16:1 n7** (Palmitoleic Acid)	6.78	8.10	8.33
**C18:0** (Stearic Acid)	3.89	5.21	5.70
**C18:1 n9 cis** (Oleic Acid)	5.31	7.09	7.33
**C18:1 n7** (Vaccenic acid)	3.03	3.85	3.99
**C18:2 n-6 cis** (Linoleic Acid)	1.40	1.14	0.88
**C18:4 n3** (Stearidonic acid)	1.61	0.66	0.30
**C20:1 n9** (Gondoic Acid)	3.17	3.69	3.84
**C20:3 n3** (Dihomo-α-linolenic Acid)	0.75	0.30	0.17
**C20:5 n-3** (Eicosapentaenoic acid EPA)	9.32	3.38	1.33
**C22:6 n-3** (Docosahexaenoic Acid DHA)	14.78	5.96	2.55
**Saturated Fatty Acids**	33.99	46.34	49.56
**Monounsaturated Fatty acids**	26.10	31.87	33.27
**Polyunsaturated Fatty acids**	29.81	12.77	6.14
**Σ n-3**	27.57	10.82	4.60
**Σ n-6**	2.23	1.94	1.55
**Σ n-3/Σ n-6**	12.34	5.57	2.97
**EPA+DHA**	24.10	9.34	3.88
**EPA/DHA**	0.63	0.57	0.52
**DHA/EPA**	1.59	1.76	1.93

Table S11. Comparison of European pilchard (*Sardina pilchardus)* fatty acid profile before and after 15 and 80 days of ensilaging.

FA	*S. pilchardus*	*S. pilchardus 15D*	*S. pilchardus 80D*
**C14:0** (Myristic Acid)	6.15	8.60	8.78
**C16:0** (Palmitic Acid)	25.54	35.10	35.93
**C16:1 n7** (Palmitoleic Acid)	5.37	6.85	6.66
**C18:0** (Stearic Acid)	5.64	7.49	7.89
**C18:1 n9 cis** (Oleic Acid)	8.13	9.98	10.00
**C18:1 n7** (Vaccenic acid)	2.72	3.48	3.49
**C18:2 n-6 cis** (Linoleic Acid)	1.69	1.26	0.82
**C18:4 n3** (Stearidonic acid)	1.32	0.42	0.25
**C20:1 n9** (Gondoic Acid)	0.95	1.10	1.17
**C20:3 n3** (Dihomo-α-linolenic Acid)	0.86	0.30	0.19
**C20:5 n-3** (Eicosapentaenoic acid EPA)	7.26	1.99	0.97
**C22:6 n-3** (Docosahexaenoic Acid DHA)	15.68	4.46	2.48
**Saturated Fatty Acids**	41.22	56.25	58.00
**Monounsaturated Fatty acids**	19.64	24.44	24.36
**Polyunsaturated Fatty acids**	29.41	10.09	6.10
**Σ n-3**	26.49	7.67	4.26
**Σ n-6**	2.93	2.41	1.84
**Σ n-3/Σ n-6**	9.06	3.18	2.32
**EPA+DHA**	22.94	6.45	3.45
**EPA/DHA**	0.46	0.45	0.39
**DHA/EPA**	2.16	2.24	2.56

Table S12. Comparison of the mix of bogue (*Boops boops*), European anchovy (*Engraulis encrasicolus*), and European pilchard (*Sardina pilchardus*) fatty acid profile before and after 15 and 80 days of ensilaging.

FA	Mix HCMR	Mix HCMR 15D	Mix HCMR 80D
**C14:0** (Myristic Acid)	6.51	9.33	9.29
**C16:0** (Palmitic Acid)	22.52	32.25	32.41
**C16:1 n7** (Palmitoleic Acid)	6.13	7.81	7.65
**C18:0** (Stearic Acid)	4.91	7.04	7.19
**C18:1 n9 cis** (Oleic Acid)	6.95	9.21	9.12
**C18:1 n7** (Vaccenic acid)	2.87	3.69	3.71
**C18:2 n-6 cis** (Linoleic Acid)	1.54	1.01	0.82
**C18:4 n3** (Stearidonic acid)	1.45	0.38	0.30
**C20:1 n9** (Gondoic Acid)	2.11	2.24	2.35
**C20:3 n3** (Dihomo-α-linolenic Acid)	0.91	0.28	0.22
**C20:5 n-3** (Eicosapentaenoic acid EPA)	8.20	2.07	1.22
**C22:6 n-3** (Docosahexaenoic Acid DHA)	15.43	4.35	2.77
**Saturated Fatty Acids**	36.82	53.04	53.33
**Monounsaturated Fatty acids**	23.25	28.72	29.00
**Polyunsaturated Fatty acids**	29.81	9.26	6.48
**Σ n-3**	27.26	7.35	4.81
**Σ n-6**	2.55	1.90	1.67
**Σ n-3/Σ n-6**	10.68	3.86	2.89
**EPA+DHA**	23.63	6.42	3.99
**EPA/DHA**	0.53	0.48	0.44
**DHA/EPA**	1.88	2.10	2.27

Table S13. Comparison of the mix of Mediterranean unsold fish species fatty acid profile before and after 80 days of ensilaging.

FA	Mix Discards	Mix Discards 80D
**C14:0** (Myristic Acid)	5.71	6.22
**C16:0** (Palmitic Acid)	24.37	28.66
**C16:1 n7** (Palmitoleic Acid)	4.39	2.99
**C18:0** (Stearic Acid)	7.88	11.91
**C18:1 n9 cis** (Oleic Acid)	12.74	10.09
**C18:1 n7** (Vaccenic acid)	2.64	2.84
**C18:2 n-6 cis** (Linoleic Acid)	1.62	0.98
**C18:4 n3** (Stearidonic acid)	0.72	0.10
**C20:1 n9** (Gondoic Acid)	0.93	0.63
**C20:3 n3** (Dihomo-α-linolenic Acid)	1.56	1.59
**C20:5 n-3** (Eicosapentaenoic acid EPA)	3.49	1.24
**C22:6 n-3** (Docosahexaenoic Acid DHA)	12.07	6.51
**Saturated Fatty Acids**	42.49	55.88
**Monounsaturated Fatty acids**	23.27	26.32
**Polyunsaturated Fatty acids**	21.78	5.96
**Σ n-3**	18.68	3.99
**Σ n-6**	3.10	1.97
**Σ n-3/Σ n-6**	6.02	2.02
**EPA+DHA**	15.56	7.75
**EPA/DHA**	0.29	0.19
**DHA/EPA**	3.46	5.25

Table S14. Comparison of bogue (*Boops boops)* mineral profile before and after 80 days of ensilaging.

Minerals & Metals	*B. boops*	*B. boops* Sil
Zn (mg/kg)	91.6	56.8
Fe (mg/kg)	86.6	87.7
Mn (mg/kg)	5.32	6.92
Ca (g/100g)	2.79	3.05
Mg (g/100g)	0.23	0.19
P (g/100g)	2.45	1.84

Table S15. Comparison of European anchovy (*Engraulis encrasicolus)* mineral profile before and after 80 days of ensilaging.

Minerals & Metals	*E. encrasicolus*	*E. encrasicolus* Sil
Zn (mg/kg)	83.0	72.6
Fe (mg/kg)	69.0	65.6
Mn (mg/kg)	9.59	9.96
Ca (g/100g)	1.75	1.46
Mg (g/100g)	0.16	0.14
P (g/100g)	1.45	1.28

Table S16. Comparison of European pilchard (*Sardina pilchardus)* mineral profile before and after 80 days of ensilaging.

Minerals & Metals	*S. pilchardus*	*S. pilchardus* Sil
Zn (mg/kg)	119	86.7
Fe (mg/kg)	117	85.7
Mn (mg/kg)	12.7	9.05
Ca (g/100g)	2.07	1.81
Mg (g/100g)	0.16	0.13
P (g/100g)	1.84	1.43

Table S17. Comparison of the mix of bogue (*Boops boops),* European anchovy (*Engraulis encrasicolus)* and European pilchard (*Sardina pilchardus)* mineral profile before and after 80 days of ensilaging.

Minerals & Metals	Mix HCMR	Mix HCMR Sil
Zn (mg/kg)	104	82.9
Fe (mg/kg)	99.8	88.8
Mn (mg/kg)	11.2	7.81
Ca (g/100g)	2.54	2.11
Mg (g/100g)	0.19	0.16
P (g/100g)	2.01	1.76

Table S18. Comparison of the mix of Mediterranean unsold fish species mineral profile before and after 80 days of ensilaging.

Minerals & Metals	Mix Discards	Mix Discards Sil
Zn (mg/kg)	75.6	81.6
Fe (mg/kg)	39.8	39.8
Mn (mg/kg)	4.97	4.30
Ca (g/100g)	3.41	3.02
Mg (g/100g)	0.17	0.13
P (g/100g)	2.44	2.28

Table S19. Mediterranean unsold fish species amino acid profile.

Essential Amino Acids (g/100g)								
Amino Acids	*Boops boops*	*Engraulis encrasicolus*	*Sardina pilchardus*	*Trachurus trachurus*	*Scomber scombrus*	*Dentex macrophthalmus*	*Pagellus erythrinus*	*Diplodus sargus*
Lysine	5.47	4.70	4.69	5.82	6.00	4.77	5.18	5.88
Methionine	1.94	1.72	1.56	2.01	2.06	1.65	1.78	2.04
Histidine	1.93	2.99	1.90	2.16	2.82	1.63	1.76	1.56
Isoleucine	2.79	2.60	2.22	2.80	2.96	2.22	2.43	2.89
Leucine	4.88	4.42	3.91	4.87	5.22	4.01	4.34	5.06
Phenylalanine	2.53	2.31	2.09	2.58	2.74	2.15	2.31	2.63
Threonine	2.91	2.57	2.33	2.91	3.24	2.46	2.67	3.03
Valine	3.11	3.04	2.55	3.10	3.46	2.75	2.84	3.21
Arginine	3.92	3.05	2.89	4.01	3.98	3.66	3.75	4.11
**Non Essential Amino Acids (g/100g)**								
Amino Acids	*Boops boops*	*Engraulis encrasicolus*	*Sardina pilchardus*	*Trachurus trachurus*	*Scomber scombrus*	*Dentex macrophthalmus*	*Pagellus erythrinus*	*Diplodus sargus*
Taurine	0.95	0.60	0.66	0.71	0.65	0.73	0.67	0.64
Tyrosine	1.76	1.65	1.38	1.77	2.15	1.54	1.74	1.93
Cysteine	0.33	0.32	0.27	0.33	0.36	0.28	0.32	0.33
Hydroxyproline	0.95	0.29	0.44	0.79	0.58	1.15	0.99	1.02
Serine	3.02	2.55	2.36	2.98	3.14	2.78	2.86	3.03
Alanine	4.22	3.75	3.20	4.21	4.13	3.82	3.90	4.38
Proline	3.03	2.20	2.11	2.87	2.77	3.03	2.94	3.13
Glutamic Acid	9.69	8.00	7.35	9.53	9.76	8.12	8.61	10.24
Aspartic Acid	6.36	5.53	5.16	6.52	6.61	5.49	5.83	6.66
Glycine	4.89	3.15	3.14	4.47	4.11	5.00	4.72	4.96

European and Greek national legislative framework that governs the management of animal (including fish) by-products.

1. Regulation (EC) 1069/2009 of the European Parliament and of the Council "on health rules for animal by-products and derived products not intended for human consumption and for the repeal of Regulation 1774/2002" (basic regulation on animal by-products) [1]

2. Commission Regulation (EU) No 142/2011 of 25 February 2011 implementing Regulation (EC) No 1069/2009 of the European Parliament and of the Council laying down health rules as regards animal by-products and derived products not intended for human consumption and implementing Council Directive 97/78/EC as regards certain samples and items exempt from veterinary checks at the border under that Directive Text with EEA relevance [2].

3. Presidential Decree 211/2006 (A211) "Supplementary measures for the implementation of Regulation 1774/2002/EC for the establishment of health rules regarding animal by-products not intended for human consumption" [3].

Food retail businesses include supermarkets that operate either independently or within supermarkets and sell their products directly to the consumer (retail sale).

The responsibility for the proper management of animal by-products produced in each food retail business concerns the entire route of the animal by-products, from the moment of their production (point of origin) to their delivery to the first approved or registered destination for use or disposal, as appropriate of the category and type of material.

In Greece, according to the Guide for the Management of Animal By-products in Super Markets (2018), retail stores with production of small quantities of category 3 materials (Reg. 1069/2009/EC, article 10, point f), up to 20 kg per week, have the possibility to dispose of these materials by other means provided that they do not present a risk to public and animal health.

The disposal in this case takes place in an approved landfill based on environmental permits for the receipt of the specific animal by-products (Reg. 1069/2009, article 19, point 1.d, Reg. 2015/9/EU, article 1, point 1 and Regulation 142/2011, Annex VI, Chapter IV)[1].

According to the Guide for the Management of Animal By-products in Super Markets 2018, by way of derogation from Regulation 1069/2009/EC, article 14 and especially the former foods of category 3 (Reg. 1069/2009/EC, article 10, point f)[1], can be disposed of in an approved landfill site (landfill), which based on environmental conditions can receive the specific ZYP, upon approval by the competent authority (Reg. 142/2011/EC, article 7) [2], and with the condition that the following apply simultaneously to the materials to be disposed of:

a) have undergone processing (Reg. (EC) 852/2004, article 2, paragraph 1, element m) [4].

b) have not been in contact with another animal by-product of category 1 or category 2 nor with another unprocessed species of category 3

c) the disposal of these materials does not pose a risk to public or animal health.

References

1. Regulation (EC) No 1069/2009 of the European Parliament and of the Council of 21 October 2009 laying down health rules as regards animal by-products and derived products not intended for human consumption and repealing Regulation (EC) No 1774/2002 (Animal by-products Regulation). *Document 32009R1069* **2009**, p. 1–33.
2. Commission Regulation (EU) No 142/2011 of 25 February 2011 implementing Regulation (EC) No 1069/2009 of the European Parliament and of the Council laying down health rules as regards animal by-products and derived products not intended for human consumption and implementing Council Directive 97/78/EC as regards certain samples and items exempt from veterinary checks at the border under that Directive Text with EEA relevance. *Document 32011R0142* **2011**, p. 1–254.
3. Presidential Decree 211/2006 (A211) "Supplementary measures for the implementation of Regulation 1774/2002/EC for the establishment of health rules regarding animal by-products not intended for human consumption". **2006**.
4. Regulation (EC) No 852/2004 of the European Parliament and of the Council of 29 April 2004 on the hygiene of foodstuffs. **2004**.
